# Population levels of sport participation: implications for sport policy

**DOI:** 10.1186/s12889-016-3463-5

**Published:** 2016-08-09

**Authors:** R. M. Eime, J. T. Harvey, M. J. Charity, W. R. Payne

**Affiliations:** 1 Institute of Sport, Exercise and Active Living, Victoria University, PO Box 14428, Melbourne, VIC 8001 Australia; 2 School of Health Sciences and Psychology, Federation University Australia, PO Box 663, Ballarat, VIC 3353 Australia

**Keywords:** Sport, Participation, Retention, Policy

## Abstract

**Background:**

Participation in sport can contribute to health-enhancing levels of leisure-time physical activity. There are recent reports that participation in sport in Australia is decreasing. However, these studies are limited to ages 15 years and over.

**Methods:**

This study integrates sports club membership data from five popular team sports and investigates sport participation across the lifespan (4–100 years) by sex and region (metropolitan/non-metropolitan).

**Results:**

Overall participant numbers per annum increased from 414,167 in 2010 to 465,403 in 2012 corresponding to a rise in the proportion of Victorian’s participating in these sports from 7.5 % in 2010 to 8.3 % in 2012. The highest proportion of participants was in the 10–14 year age range, with participation rates of 36 % in 2010 and 40 % in 2012. There was a considerably lower participation rate in the 15–19 year age group compared to the 10–14 age group, in all three years studied, and the decline continued progressively with increasing age. Male and female age profiles of participation were generally similar in shape, but the female peak at age 10–14 was sharper than for the males, and conversely there were very few 4 year old female participants. Participation rates were generally higher in non-metropolitan than metropolitan areas; the difference increased with increasing age from 4 to 34 years, then steadily declined, reaching parity at around 60 years of age.

**Conclusions:**

It is a positive sign that participation in these popular sports increased by over 50,000 participants from 2010 to 2012. Large proportions of the population aged 5–14 participate in club based sport. Participation rates decline sharply in late adolescence, particularly for females, and while this may not be a concern from a broad health perspective so long as they transition into other forms of physical activity, it is certainly a matter of concern for the sport sector. It is recommended that sport policy places a higher priority on grass-roots participation and that sporting organisations are supported to prioritise the retention issues occurring during adolescence, particularly for females so as to maximise the potential for sport to maintain its positive contribution to population wellbeing.

## Background

Understanding population sport participation is recommended to inform evidence-based strategic planning and policy development of sport [1–3]. Participation in sport can contribute positively to a range of physical, mental and social health dimensions [4, 5]. Furthermore, people who maintain participation in sport throughout childhood reportedly have higher health-related quality of life compared to those who do not participate in sport [6], and are more likely to be active as adults [7–9]. On the other hand, it is acknowledged that not everyone is attracted to sport, and that people can be active through other leisure-time pursuits [10]. Furthermore, sport is not always inherently healthy, as it has been associated with an increased risk of a range of detrimental effects, including injury [11], body image issues [12] and negative aspects of the focus on competition [13].

Nevertheless, notwithstanding these risks, on balance the contribution of sport participation to public health is generally evidenced in the research literature [4, 5] and consequently the measurement of sport participation and knowledge about trends in participation is important for a range of sectors and organisations, including sport governing bodies, government sports agencies, as well as sport and recreation and health organisations [3]. Corresponding with this, a whole-of-government approach to the investment in sport planning for programs and facilities has been recently advocated [14]. Information on sport trends could provide the evidence to inform policy and strategic investments, however there is little knowledge about participation in club-based sport [3]. High quality sport participation data is required to provide the evidence to inform development of sport programs and policies to meet community needs [3].

Both age and gender are important factors influencing participation in sport [15]. Participation in sport is popular especially amongst children and young adolescents [16–18]. However there are reports of sport participation decreasing during adolescence and continuing to decrease throughout adulthood [16, 19, 20]. A recent study reported that whilst physical activity (PA) levels in general did not decline during adolescence for females the contexts of participation changed [21]. Older adolescent females (16–18 years) shifted their PA participation away from organised, competitive modes and settings, towards non-organised and non-competitive modes and settings. This corresponded to a change from team-based sport activities to individual types of PA [21]. Furthermore, internationally, there is consistent evidence that males are more likely to participate in sport than females [3, 22]. Therefore, examining sport participation across the lifespan is important given the significant differences in participation by age and gender.

Studies examining sport participation are often based on an age-limited sample of participants, such as those aged 15 years and older [3, 22], and the findings from studies using small samples are often not able to be extrapolated to population based levels with high degrees of accuracy. Furthermore, many studies are hindered by low recruitment rates, meaning that they may recruit a biased sample of people who are already interested in PA [23].

It has been recently suggested that the integration of sports participation data from multiple sources could provide a sector-wide understanding of participation as a means to provide a strong evidence base to inform policy development [3]. This study aims to understand participation across the lifespan in five popular sports in the Australian state of Victoria, by sex and region, over the 3-year period 2010–2012.

## Methods

### Study design

This study draws on the participant membership records from five sports (Australian rules football, basketball, cricket, hockey and netball), in the Australian state of Victoria for the period 2010–2012. A participant was defined as a registered member of a club affiliated with their sport’s governing body. Four of the five sports were ranked within the top 10 organised physical activities and regular club-based physical activities in Australia for persons aged 15 years and over [24] and within the top 10 organised physical activities for children aged 5–14 years [25].

### Statistical analysis

Data for the five sports were analysed together to create broadly based participation profiles while maintaining confidentiality of membership data for individual sports. An individual could engage in more than one sport and was counted separately in each sport. As such, counts of participants are somewhat inflated, and can be considered to be weighted by the number of the five sports participated in by each individual, i.e. weighted to some extent by individuals’ levels of sport participation. The reported rates are strictly ‘registrations per 100 persons’ but for brevity and simplicity they are referred to throughout as percentages of the relevant population cohort.

Age-specific participation rates (or prevalences) were calculated for standard 5-year cohorts defined by the Australian Bureau of Statistics (ABS), by dividing the number of participants in each age cohort by the corresponding estimated resident population (ERP) [26]. The reference population for 4 year-old participants was estimated as a proportion of the 0–4 cohort, calculated using single-year-of-age breakdowns from the 2011 population census [27]. Separate age-specific participation profiles were also calculated for each sex and for two regions - metropolitan Melbourne and non-metropolitan Victoria. Regional breakdowns were based on residential postcodes, with categorisation into metropolitan and non-metropolitan regions defined by ABS [28]. Approval to undertake the study was granted by the Human Research Ethics Committee of Federation University, Australia.

Age profiles of overall participation were graphed for the three years 2010–2012, together with age profiles by sex and region for the year 2012. Rate (or prevalence) ratios and associated confidence intervals were used to make comparisons between age-specific participation rates for different years, age groups, sexes, and regions. Analyses were conducted using Microsoft Excel and SPSS Version 21.

## Results

In 2012, a total of 465,403 individuals participated in the five sports studied. Two of the five sports included in the study (Australian Rules Football and cricket) were male-dominated (>95 % males), one (netball) was female dominated (>95 % females) and two (basketball and hockey) had around two-thirds male and one-third female participants. Consequently, a greater proportion of the study population were males (*n* = 320,842; 68.9 %) than females (*n* = 144,561; 31.1 %).

**Fig. 1:**
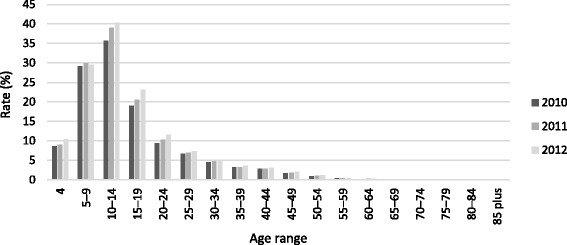
Age-specific participation rates 2010–2012

**Fig. 2:**
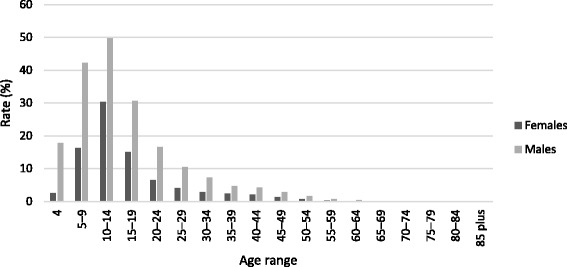
Age-specific participation rates 2012: comparisons by sex

**Fig. 3:**
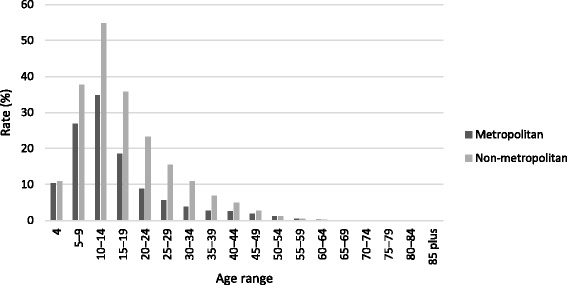
Age-specific participation rates 2012: comparisons by region

Table 1 and Fig. 1 show age-specific participation rates over the period 2010–2012. Table 1 also shows comparisons between 2011 and 2010 and between 2012 and 2011, in the form of rate ratios and associated confidence intervals. Table 2 shows, for the year 2012, the participation rate for each age group compared to the 10–14 year-old age group, which is when the participation rate peaks. Table 3 and Fig. 2 show differences in 2012 age-specific participation rates for females compared to males. Table 4 and Fig. 3 show differences in 2012 age-specific participation rates for the non-metropolitan region compared to the metropolitan region.

**Table 1:** Age-specific participation rates 2010–2012: comparisons by year

	2010			2011			2012			2011 v 2010			2012 v 2011		
Age range	Count^a^	ERP^b^	Rate^c^	Count^a^	ERP^b^	Rate^c^	Count^a^	ERP^b^	Rate^c^	Ratio^d^	95 % CI		Ratio^d^	95 % CI	
4	6,212	71,543	8.7	6,443	71,371	9.0	7,594	72,820	10.4	1.04	1.01	1.07	1.16	1.13	1.18
5–9	95,765	328,103	29.2	99,510	332,729	29.9	100,787	340,503	29.6	1.02	1.02	1.03	0.99	0.98	1.00
10–14	120,039	335,650	35.8	128,644	330,056	39.0	132,965	330,448	40.2	1.09	1.08	1.10	1.03	1.03	1.04
15–19	69,641	365,388	19.1	72,711	354,583	20.5	81,910	355,117	23.1	1.08	1.07	1.08	1.12	1.12	1.13
20–24	39,768	423,668	9.4	42,628	412,447	10.3	47,831	412,009	11.6	1.10	1.09	1.11	1.12	1.11	1.13
25–29	28,364	423,593	6.7	29,560	424,794	7.0	31,965	433,533	7.4	1.04	1.03	1.05	1.06	1.05	1.07
30–34	17,569	390,883	4.5	18,579	391,818	4.7	20,529	406,284	5.1	1.05	1.04	1.07	1.07	1.05	1.08
35–39	13,179	407,231	3.2	12,973	395,277	3.3	13,952	391,179	3.6	1.01	1.00	1.03	1.09	1.07	1.11
40–44	11,251	392,290	2.9	11,443	400,351	2.9	12,831	411,667	3.1	1.00	0.98	1.02	1.09	1.07	1.11
45–49	6,872	386,840	1.8	6,839	379,272	1.8	7,824	378,012	2.1	1.02	0.99	1.04	1.15	1.12	1.17
50–54	3,434	360,399	1.0	3,659	363,823	1.0	4,357	371,333	1.2	1.06	1.02	1.09	1.17	1.13	1.20
55–59	1,175	322,839	0.4	1,280	325,633	0.4	1,570	333,160	0.5	1.08	1.02	1.14	1.20	1.14	1.26
60–64	565	295,613	0.2	673	297,597	0.2	789	296,984	0.3	1.18	1.09	1.28	1.17	1.09	1.26
65–69	189	222,468	0.1	195	232,880	0.1	288	249,830	0.1	0.99	0.85	1.15	1.38	1.21	1.56
70–74	105	178,634	0.1	123	181,754	0.1	144	188,531	0.1	1.15	0.95	1.40	1.13	0.94	1.35
75–79	12	141,720	<0.1	17	143,135	<0.1	33	146,349	<0.1	1.40	0.74	2.66	1.90	1.20	3.00
80–84	13	114,211	<0.1	9	115,327	<0.1	12	114,990	<0.1	0.69	0.31	1.53	1.34	0.61	2.93
85–100	14	102,884	<0.1	16	104,390	<0.1	22	110,096	<0.1	1.13	0.60	2.11	1.30	0.76	2.24
All	414,167	5,545,932	7.5	435,302	5,537,817	7.9	465,403	5,629,122	8.3	1.05	1.05	1.06	1.05	1.05	1.05

^a^Membership registrations in five sports

^b^Estimated resident population

^c^Membership registrations per 100 persons

^d^Rate ratio

**Table 2:** Age-specific participation rates 2012: comparisons by age

Age range	Count^a^	ERP^b^	Rate^c^	Ratio^d^	95 % CI	
4	7,594	72,820	10.4	0.26	0.26	0.26
5–9	100,787	340,503	29.6	0.74	0.74	0.74
10–14	132,965	330,448	40.2	1	1	1
15–19	81,910	355,117	23.1	0.57	0.57	0.57
20–24	47,831	412,009	11.6	0.29	0.29	0.29
25–29	31,965	433,533	7.4	0.18	0.18	0.18
30–34	20,529	406,284	5.1	0.13	0.13	0.13
35–39	13,952	391,179	3.6	0.09	0.09	0.09
40–44	12,831	411,667	3.1	0.08	0.08	0.08
45–49	7,824	378,012	2.1	0.05	0.05	0.05
50–54	4,357	371,333	1.2	0.03	0.03	0.03
55–59	1,570	333,160	0.5	0.01	0.01	0.01
60–64	789	296,984	0.3	0.01	0.01	0.01
65–69	288	249,830	0.1	<0.01	<0.01	<0.01
70–74	144	188,531	0.1	<0.01	<0.01	<0.01
75–79	33	146,349	<0.1	<0.01	<0.01	<0.01
80–84	12	114,990	<0.1	<0.01	<0.01	<0.01
85–100	22	110,096	<0.1	<0.01	<0.01	<0.01
All	465,403	5,629,122	8.3	0.21	0.21	0.21

^a^Membership registrations in five sports

^b^Estimated resident population

^c^Membership registrations per 100 persons

^d^Rate ratio: reference category = 10–14

**Table 3:** Age-specific participation rates 2012: comparisons by sex

	Female			Male			Female v Male		
Age range	Count^a^	ERP^b^	Rate^c^	Count^a^	ERP^b^	Rate^c^	Ratio^d^	95 % CI	
4	905	35,368	2.6	6,689	37,452	17.9	0.14	0.13	0.15
5–9	27,003	165,732	16.3	73,784	174,771	42.2	0.39	0.38	0.39
10–14	48,808	161,124	30.3	84,157	169,324	49.7	0.61	0.60	0.61
15–19	26,101	173,194	15.1	55,809	181,923	30.7	0.49	0.49	0.50
20–24	13,022	202,220	6.4	34,809	209,789	16.6	0.39	0.38	0.39
25–29	8,906	215,027	4.1	23,059	218,506	10.6	0.39	0.38	0.40
30–34	5,771	203,242	2.8	14,758	203,042	7.3	0.39	0.38	0.40
35–39	4,841	197,185	2.5	9,111	193,994	4.7	0.52	0.51	0.54
40–44	4,420	209,300	2.1	8,411	202,367	4.2	0.51	0.49	0.52
45–49	2,594	192,101	1.4	5,230	185,911	2.8	0.48	0.46	0.50
50–54	1,325	188,505	0.7	3,032	182,828	1.7	0.42	0.40	0.45
55–59	499	169,629	0.3	1,071	163,531	0.7	0.45	0.41	0.49
60–64	220	151,622	0.1	569	145,362	0.4	0.37	0.32	0.42
65–69	73	127,478	0.1	215	122,352	0.2	0.33	0.26	0.41
70–74	40	96,918	<0.1	104	91,613	0.1	0.36	0.26	0.51
75–79	16	78,717	<0.1	17	67,632	<0.1	0.81	0.44	1.48
80–84	4	65,559	<0.1	8	49,431	<0.1	0.38	0.11	1.28
85–100	13	71,354	<0.1	9	38,742	<0.1	0.78	0.37	1.68
All	144,561	2,843,674	5.1	320,842	2,785,448	11.5	0.44	0.44	0.44

^a^Membership registrations in five sports

^b^Estimated resident population

^c^Membership registrations per 100 persons

^d^Rate ratio

**Table 4:** Age-specific participation rates 2012: comparisons by region

	Non-metropolitan			Metropolitan			Non-metro v Metro		
Age range	Count^a^	ERP^b^	Rate^c^	Count^a^	ERP^b^	Rate^c^	Ratio^d^	95 % CI	
4	1,980	18,126	10.9	5,614	54,666	10.3	1.06	1.02	1.11
5–9	32,597	86,316	37.8	68,190	254,187	26.8	1.41	1.39	1.42
10–14	48,818	89,011	54.8	84,147	241,437	34.9	1.57	1.56	1.59
15–19	33,404	93,232	35.8	48,506	261,885	18.5	1.93	1.91	1.96
20–24	18,738	80,650	23.2	29,093	331,359	8.8	2.65	2.61	2.68
25–29	11,918	76,624	15.6	20,047	356,909	5.6	2.77	2.72	2.82
30–34	8,039	74,223	10.8	12,490	332,061	3.8	2.88	2.82	2.94
35–39	5,532	81,096	6.8	8,420	310,083	2.7	2.51	2.45	2.58
40–44	4,572	92,550	4.9	8,259	319,117	2.6	1.91	1.85	1.97
45–49	2,494	92,505	2.7	5,330	285,507	1.9	1.44	1.39	1.50
50–54	1,187	98,713	1.2	3,170	272,620	1.2	1.03	0.98	1.10
55–59	448	94,519	0.5	1,122	238,641	0.5	1.01	0.92	1.11
60–64	219	88,594	0.2	570	208,390	0.3	0.90	0.79	1.04
65–69	78	76,448	0.1	210	173,382	0.1	0.84	0.67	1.06
70–74	28	58,301	<0.1	116	130,230	0.1	0.54	0.37	0.79
75–79	9	44,610	<0.1	24	101,739	<0.1	0.86	0.41	1.78
80–84	5	34,830	<0.1	7	80,160	<0.1	1.64	0.52	5.23
85–100	0	32,781	0.0	22	77,315	<0.1	0.00		
All	170,066	1,380,778	12.3	295,337	4,248,344	7.0	1.77	1.76	1.78

^a^Membership registrations in five sports

^b^Estimated resident population

^c^Membership registrations per 100 persons

^d^Rate ratio

Overall, the number of registered participants in the five sports studied rose from 414,167 in 2010 to 465,403 in 2012. This corresponded to a rise in the proportion of Victorians participating in these sports, from 7.5 % in 2010 to 8.3 % in 2012.

Table 1 and Fig. 1 reveal the following patterns and trends. The rate of registered sport participation among 4 year olds rose from 8.7 % in 2010 to 9.0 % in 2011, and then increased quite sharply to 10.4 % in 2012. By contrast, the 5–9 year age cohort had the second highest proportion of registered sport participants, but this proportion remained very stable throughout 2010–2012 at just under 30 %. The highest proportion of registered sport participants in the Victorian population occurred within the 10–14 year age range, and this increased considerably between during the period from 35.8 % in 2010 to 39.0 % in 2011 and 40.2 % in 2012. In each year there was a considerable decline in the participation rate with increasing age from 10–14 to 15–19 and 20–24. However, over the three year period, these three age cohorts all exhibited strong growth in participation. Beyond age 24, the participation rate declined steadily with increasing age, and as the size of the counts diminished, the rate ratios in Table 1 became more volatile.

Table 2 presents the age-specific participation rates for the year 2012 from a different perspective, with the rate for each cohort being compared with the 10–14 cohort, the age of peak participation. For example, Table 2 shows that the participation rate for 20–24 year olds in 2012 was 11.6 %, which was 0.29 (or 29 %) of the peak participation rate of 40.2 %.

Table 3 and Fig. 2 show that more males than females were registered in 2012 as participants in the five sports included in this study, and this was consistently the case for each age cohort. The overall male participation rate was 11.5 %, compared to 5.1 % for females. Participation rates peaked at age 10–14 for both males (49.7 %) and females (30.3 %). Excluding some of the oldest age cohorts for which numbers were very small, the highest rate ratio (0.61) was for the 10–14 age cohort, indicating that this is the age at which the female participation rate comes closest (at 61 %) to the male participation rate. The lowest rate ratio was for 4-year olds (males 17.9 %; females 2.6, or 14 % of the male rate).

Table 4 and Fig. 3 show that the overall participation rate in the five sports studied was higher in non-metropolitan (12.3 %) compared to metropolitan areas (7.0 %). Among 4 year olds, the rates were similar (rate ratio = 1.06), but the rate ratio steadily increased with increasing age, reaching a maximum of 2.88 at age 30–34. Thereafter, the rate ratio declined, reaching parity around age 60. Participation rates peaked at age 10–14 for both metropolitan (34.9 %) and non-metropolitan (54.8 %) regions.

## Discussion

This study uniquely provides details of population-level participation in sport across a whole state, across the lifespan, over a 3-year period, based on over 400,000 participant registrations in five popular sports. It provides evidence of the trends in participation across the lifespan for both sexes and for metropolitan and non-metropolitan regions. The summaries presented in this paper, and the more detailed multi-factorial profiles which can be constructed from these data, can be utilised by the sport sector and government for planning of policy and strategic directions to increase participation in sport. This paper contributes to an identified need for more comprehensive sport participation data to inform evidence-based decision making [3].

The overall participation rate for the five sports included in this study increased from 7.5 to 8.3 % over the three year period. It is difficult to compare this to other studies, which are generally more broadly based, although generally age-limited, and which often employ more inclusive definitions of participation. For instance, Eime, Sawyer et al. (2014) reported participation rates for the period 2001–2010 of around 30 % for males and 20 % for females aged 15 years and above, but included all sporting activity organised by a sport or recreation club or association, and defined participation as having participated at least once in the preceding 12 months. This study also reported no significant changes in sport participation rates from 2001 to 2010 [3]. Another study reported that for Australians aged 15 years and over, participation in any form of “sport and physical recreation” at least once in the preceding 12 months decreased from 65 % in 2011–2012 to 60 % in 2013–2014 [29]. The current study is more specifically focused on club sport per se, and while based on only five sports, extends the findings of other studies in that it captures sport participation across the lifespan, and in particular includes children, for whom sport is a popular activity, Further, this study is unique as it is based on a census of sport club members and is therefore inherently more accurate than a cross sectional sample survey.

Over a third of all Victorians aged 10–14 years were registered participants in these five sports and the participation rate increased by four percentage points between 2010 (36 %) and 2012 (40 %). This was the age range with the highest participation rate, which is consistent with the study of Olds et al., [18], which reported peak sport participation at age 12–13 years. The second highest participation rate was among the 5–9 year olds, which was 30 % for each of the three years.

Whilst a large proportion of the population plays sport in childhood, this declines rapidly in late adolescence. The present study showed that in 2012 there was a decline of 17 percentage points in the rate of participation from 10–14 to 15–19 years. Other studies of organised sport participation have reported that 60 % of children aged 5–14 years participated in at least one organised sport outside of school hours [16] compared with only 28 % aged 15 years and older [29]. This decline in participation during adolescence is partly explained by a change in context of participation in PA away from club- and team-based participation towards unorganised and individual PA pursuits [21, 30]. By age 20 only 12 % participate in these sports, with further declines with age evident across the lifespan.

Furthermore, this decline coincides with the age when elite pathways open up through talent programs, and a small minority of participants move to national and then international competitions [31]. However the impact of the more broadly-based drop-off in sport participation during adolescence on the health of individuals and communities is not known [21].

While it is a consistent finding that males are more likely to participate in sport than females [18, 30], the present study does not contribute to such comparisons because of the gender bias in the particular five sports studied. However, the present study shows that the under-representation of females relative to males was greatest in the youngest age groups in those sports studied. This finding may support the contention that parents are more likely to provide sport opportunities to their sons rather than daughters [32].

Location of residence was also a factor contributing to participation in these five sports. Participation rates were much higher in non-metropolitan than metropolitan areas, for both males and females. This may be related to the sports being traditional sports which are readily available in non-metropolitan areas; whilst within metropolitan areas there is often greater choice in types of sports and recreation activities for young people [10]. The participation rate for males in non-metropolitan aged 10-14 years was particularly high, at 64 % in 2012, which was 20 percentage points higher than for metropolitan males in this age group.

### Limitations

We acknowledge some limitations to this study. The number of sports studied was limited and there was no way of detecting multiple counting of those who participated in more than one of the five sports. There was also sex bias in the data; of the five sports included, three are predominantly played by males and only one is predominantly played by females, which precludes meaningful comparison between the participation rates of males and females. However notwithstanding this, comparison of sex-specific rates of participation over time or age-specific rates of participation for each sex does not require equal representation of the sexes in the data set.

## Conclusion

It is a positive result that participation in sport has increased by over 50,000 participants from 2010 to 2012 in these popular sports.

The apparent contradiction of this result to reports that rates of participation in sport are not increasing [3] or are decreasing [29] is likely due to the inclusion of children in the whole lifespan scope of the present study, and the identification that the highest level of participation occurs in children, particularly those aged 5–14 years.

These increases are likely due to a range of policy and strategic investments from sports organisations as well as other organisations such as VicHealth and the Victorian state government. Sport is doing well at capturing large proportions of the population at an early age. However attention now needs to be given to the development of strategies for retention for those aged older than 15, and especially for females, given the domination of participation amongst males, and the greater decline in female participation rates with increasing age beyond early adolescence. We need to better understand the contribution of sport to overall PA levels, and therefore to health, across the lifespan.

Currently national funding for sport is heavily skewed towards high performance/elite sport rather than grass-roots community sport [33]. Sports are also encouraged to increase numbers of club members from year to year [34], which may encourage prioritising recruitment of new and younger participants, rather than on more specific retention strategies. Perhaps a more balanced approach and targeted policy towards population-based participation may support sport to prioritise the retention issues occurring during adolescence, particularly for females. It is recommended that sport policy focuses on maintaining participants and not simply total throughput of members each year.

Large proportions of the population aged 5–14 participate in club based sport. However the questions remain, can sport curb the attrition during late adolescence, maintaining people engaged in sport or will the trend away from sport towards other leisure-time pursuits continue?

Finally, volunteers are a vital component of the capacity and viability of sports clubs [17]. Therefore, research encompassing a broader scope of participation, not only including a wider range of sports, but also including volunteers who may be relatively inactive themselves but who are vital to facilitating the activity of others, would contribute further to the understanding of the contribution of sport for individuals and communities.

## Abbreviations

ABS, Australian bureau of statistics; ERP, Estimated resident population; PA, Physical activity.

## References

[1] Rowe K, Shilbury D, Ferkins L, Hinckson E (2013). Sport development and physical activity promotion: An integrated model to enhance collaboration and understanding. Manage Rev.

[2] Henderson KA (2009). A paradox of sport management and physical activity interventions. Sport Manage Rev.

[3] Eime R, Sawyer N, Harvey J, Casey M, Westerbeek H, Payne W (2015). Integrating Public Health and Sport Management: Sport participation trends 2001–2010. Sport Manage Rev.

[4] Eime R, Young J, Harvey J, Charity M, Payne W (2013). A systematic review of the psychological and social benefits of participation in sport for adults: Informing development of a conceptual model of health through sport. Inter J Behav Nutrn Phys Act.

[5] Eime R, Young J, Harvey J, Charity M, Payne W (2013). A systematic review of the psychological and social benefits of participation in sport for children and adolescents: informing development of a conceptual model of health through sport. Inter J Behav Nutrn Phys Act.

[6] Vella SA, Cliff DP, Magee CA, Okely AD (2014). Sports Participation and Parent-Reported Health-Related Quality of Life in Children: Longitudinal Associations. J Pediatr.

[7] Richards R, Williams S, Poulton R, Reeder A (2007). Tracking club sport participation from childhood to early adulthood. Res Q Exerc Sport.

[8] Telama R, Yang X, Hirvensalo M, Raitakari O (2006). Participation in organized youth sport as a predictor of adult physical activity: A 21-year longitudinal study. Pediatr Exerc Sci.

[9] Dohle S, Wansink B (2013). Fit in 50 years: participation in high school sports best predicts one’s physical activity after Age 70. BMC Public Health.

[10] Eime RM, Charity MJ, Harvey JT, Payne WR (2015). Participation in sport and physical activity: associations with socio-economic status and geographical remoteness. BMC Public Health.

[11] Drew MK, Finch CF (2016). The Relationship Between Training Load and Injury, Illness and Soreness: A Systematic and Literature Review. Sports Med.

[12] Slater A, Tiggemann M (2011). Gender differences in adolescent sport participation, teasing, self-objectification and body image concerns. J Adolesc.

[13] Eime RM, Casey MM, Harvey JT, Charity MJ, Young JA, Payne WR (2015). Participation in modified sports programs: a longitudinal study of children’s transition to club sport competition. BMC Public Health.

[14] Vicsport: More Victorians in the game. Vicsport policy agenda. Melbourne: Vicsport; 2014.

[15] Scheerder J, Vanreusel B, Taks M (2005). Stratification Patterns of Active Sport Involvement Among Adults: Social Change and Persistence. Inter Rev Sociol Sport.

[16] Australian Bureau of Statistics (2012). Children’s participation in cultural and leisure activities.

[17] Eime R, Payne W, Harvey J (2009). Trends in organised sport membership: Impact on sustainability. J Sci Med Sport.

[18] Olds T, Dollman J, Maher C (2009). Adolescent sport in Australia: Who, when, where and what?. ACHPER Healthy Lifestyles J.

[19] Maia JAR, Lefevre J, Claessens AL, Thomis MA, Peeters MW, Beunen GP (2010). A growth curve to model changes in sport participation in adolescent boys. Scand J Med Sci Sports.

[20] Birchwood D, Roberts K, Pollock G (2008). Explaining differences in sport participation rates among young adults: Evidence from the South Caucasus. Eur Phys Educ Rev.

[21] Eime R, Harvey J, Sawyer N, Craike M, Symons C, Polman R, Payne W (2013). Understanding the contexts of adolescent female participation in sport and physical activity. Res Q Exerc Sport.

[22] Van Tuyckom C, Scheerder J, Bracke P (2010). Gender and age inequalities in regular sports participation: A cross-national study of 25 European countries. J Sports Sci.

[23] Eime RM, Casey MM, Harvey JT, Sawyer NA, Symons CM, Payne WR (2015). Socioecological factors potentially associated with participation in physical activity and sport: A longitudinal study of adolescent girls. J Sci Med Sport.

[24] Australian Sports Commission (2010). Participation in Exercise, Recreation and Sport.

[25] Australian Bureau of Statistics (2013). Children’s participation in sport and physical recreation.

[26] Australian Bureau of Statistics (2011). Information Paper: Population Estimates under Australia’s New Statistical Geography, Cat.No.3219.0.55.001.

[27] Australian Bureau of Statistics: Census of Population and Housing: Basic Community Profile DataPack, 2011. Cat. No. 2069.0.30.008. Canberra: Australian Bureau of Statistics; 2011.

[28] Australian Bureau of Statistics (2011). Australian Statistical Geography Standard (ASGS): Volume 1- Main Structure and Greater Capital City Statistical Areas.

[29] Participation in sport and physical recreation, Australia, 2013–2014. [http://www.abs.gov.au/AUSSTATS/abs@.nsf/mediareleasesbyReleaseDate/7E9A3938BB83490CCA256DF5007C3738?OpenDocument&utm_medium=Email&utm_source=ExactTarget&utm_campaign=]. Accessed 8 Aug 2016.

[30] Vella S, Cliff D, Okely A (2014). Socio-ecological predictors of participation and dropout in organised sports during childhood. Int J Behav Nutr Phys Act.

[31] Netball Australia Athlete Pathway. http://netball.com.au/get-involved/player-pathways/. Accessed 8 Aug 2016.

[32] Eccles JS, Jacobs JE, Harold RD (1990). Gender Role Stereotypes, Expectancy Effects, and Parents’ Socialization of Gender Differences. J Soc Issues.

[33] National Sporting Organisation and National Sporting Organisation for persons with a disability. 2013–2014. funding.

[34] Australian Sports Commission (2011). Strategic Plan 2011–2012 to 2014–2015.

